# Nonoperative Management of a Spontaneous Perforation of Neobladder Secondary to Blackout From Solitary Binge Drinking: A Case Report and Current Literature Review

**DOI:** 10.7759/cureus.69749

**Published:** 2024-09-19

**Authors:** Shamik Giri, Ahmed A Ahmed, Mohamed Zeid, Muhammad S Khan, Subhasis K Giri

**Affiliations:** 1 Medicine, St. James's Hospital and Trinity College Dublin, Dublin, IRL; 2 Urology, University Hospital Limerick, Limerick, IRL; 3 Urology, King's College London, Guy's and St. Thomas' Hospitals, King's Health Partners, London, GBR; 4 Urology/Surgery, University Hospital Limerick and University of Limerick School of Medicine, Limerick, IRL

**Keywords:** timed voiding, urinary retention, cautious active monitoring, nonoperative management, spontaneous perforation, binge drinking, robotic, orthotopic neobladder

## Abstract

The orthotopic ileal neobladder is becoming a popular technique of urinary diversion after radical cystectomy (RC) for localized muscle-invasive bladder cancer (MIBC), allowing patient continence, with a more desirable body image and good quality of life.

Minimally invasive robot-assisted RC and neobladder have the potential to minimize physical and psychological trauma and are increasingly being adopted for patients with MIBC worldwide. Spontaneous perforation of orthotopic neobladder is uncommon;however, it represents serious complications. Solitary binge drinking can be dangerous in a patient with a neobladder because of reduced level of consciousness and overdistension of the neobladder. We report a case of spontaneous ileal neobladder perforations one year post-robotic RC secondary to blackouts from binge drinking. We also describe nonoperative active management and review the literature. A 66-year-old gentleman was brought by ambulance to our emergency department with a reduced level of consciousness, vomiting, and abdominal pain in the early hours of the morning. Collateral history revealed that he had drunk alcohol alone the night before at his home where he lives alone. Initial examination revealed tachycardia and hypotension. Immediate resuscitation using the sepsis six protocol included intravenous normal saline, blood culture, broad-spectrum antibiotic, lactate measurement, and insertion of a urethral catheter to monitor urine output. Following contrast-enhanced computed tomography (CECT) of the abdomen and pelvis, the patient was referred to a urologist. A diagnosis of spontaneous perforation of the neobladder was made. A nonoperative or ‘conservative’ management approach was adopted with careful active monitoring at the intensive care unit (ICU) involving a multidisciplinary team. Follow-up CT was performed to assess radiological recovery. The patient recovered successfully and was discharged home five weeks post-admission with an indwelling urethral catheter. The catheter was removed 10 weeks post-admission following a cystogram confirming the integrity of the neobladder. The patient has preserved neobladder function and continence and is doing well until the last follow-up at six months post-discharge. Patients with neobladder should be rigorously counseled about the importance of timed voiding, intermittent self-catheter, serious consequences of solitary binge drinking, and urinary retention.

## Introduction

Radical cystectomy (RC) with pelvic lymph node dissection and urinary diversion is the gold-standard therapy for localized muscle-invasive bladder cancer (MIBC). Traditionally, ileal conduit formation following RC has been the preferred type of urinary diversion. More recently, orthotopic neobladder has become increasingly popular, allowing patients with continence a more desirable body image and good quality of life [1,2]. Furthermore, minimally invasive robot-assisted RC and neobladder have the potential to minimize physical and psychological trauma and are increasingly being adopted for patients with MIBC worldwide.

The ileum is metabolically safer than other intestinal segments due to less electrolyte absorption. In addition, urinary reservoirs constructed from detubularized ileum have superior urodynamic qualities compared to other intestinal segments [2]. The main long-term complications of ileal neobladder are infections, stone formation, ureteroenteric stricture, voiding dysfunction, metabolic abnormalities, tumors, and perforations.

Spontaneous perforation of orthotopic neobladder is uncommon but a serious complication. Nippgen et al. reported a spontaneous perforation rate of 4.3% [3]. Overdistension of the neobladder is the main factor in the etiopathogenesis. Other risk factors include transmural infection, trauma, and ischemia. Presentation of patients with spontaneous perforation of the neobladder can present to an emergency department in a wide variety of ways such as abdominal discomfort, pain, distension, vomiting, or sepsis.

Other factors that might play a role in such complications include excessive alcohol intake. Drinking alcohol not only causes diuresis but also reduces the level of consciousness and thus impairs one’s ability to timely voiding. Solitary heavy drinkers are likely to have lower distress tolerance than social drinkers. Solitary binge drinking is more likely to have a greater number and severity of alcohol-related problems and depression [4]. Thus, solitary binge drinking can be dangerous in a patient with a neobladder. Timed voiding is crucial to minimize the risk of spontaneous bladder perforation. The patient should be counseled about the care of the neobladder.

Furthermore, over the years, there has been a shift from operative to increasingly nonoperative or ‘conservative’ management of abdominal visceral injuries especially for solid organs [5].

We report a case of spontaneous ileal neobladder perforations one year post-robotic RC secondary to blackouts from binge drinking. We also describe nonoperative active management and review the literature.

## Case presentation

A 66-year-old gentleman was brought by ambulance to our emergency department with a reduced level of consciousness, vomiting, and abdominal pain in the early hours of the morning. Collateral history revealed that he had drunk alcohol alone the night before at his home where he lives alone. He was found lying on the floor with abdominal pain. On arrival at the emergency department, examination revealed a respiration rate of 12 per minute, oxygen saturation (SaO2) of 91%, temperature of 35.5°C, pulse rate of 110/min, and BP of 100/60 mm of Hg.

He was resuscitated using the Sepsis Six protocol and monitored at the emergency department [6]. The Sepsis Six protocol was based on the ‘Surviving sepsis campaign’ [7]. The airway was maintained, and oxygen was administered via a nonrebreather face bag. Intravenous normal saline (0.9%) infusion was started. Blood was taken for full blood count (FBC), C-reactive protein (CRP), creatinine, lactate, and culture. Then, a broad-spectrum antibiotic piperacillin/tazobactam was administered. Size 16 Fr all silicone urinary catheter was inserted and recorded drainage of 50 ml of cloudy urine. Urine was sent for microscopy and culture. Urine microscopy revealed leucocytes of 100-200/ul, red cells of >200/ul, and the presence of bacteria (Table 1).

**Table 1:** Urine microscopy results on admission

Urine microscopy parameter	Results	Range
Urine leucocytes	100-200	<10/µL
Urine red cells	>200	<5/µL
Bacteria	Present	

On admission, blood results revealed an elevated white blood count of 12.72 x 109/L with neutrophilia, CRP of 404 mg/L, creatinine of 400 umol/L, and lactate of 3.5 mmol/L (Table 2).

**Table 2:** Blood parameters on admission

Blood parameter	Result	Reference range/units
White blood count (WBC)	12.72	4.0-11.0 x 10⁹/L
Neutrophils	10.5	2.0-7.0 x 10⁹/L
C-reactive protein (CRP)	404	0-5 mg/L
Creatinine	400	60-110 µmol/L
Lactate	3.5	0.4-0.8 mmol/L

**Figure 1:**
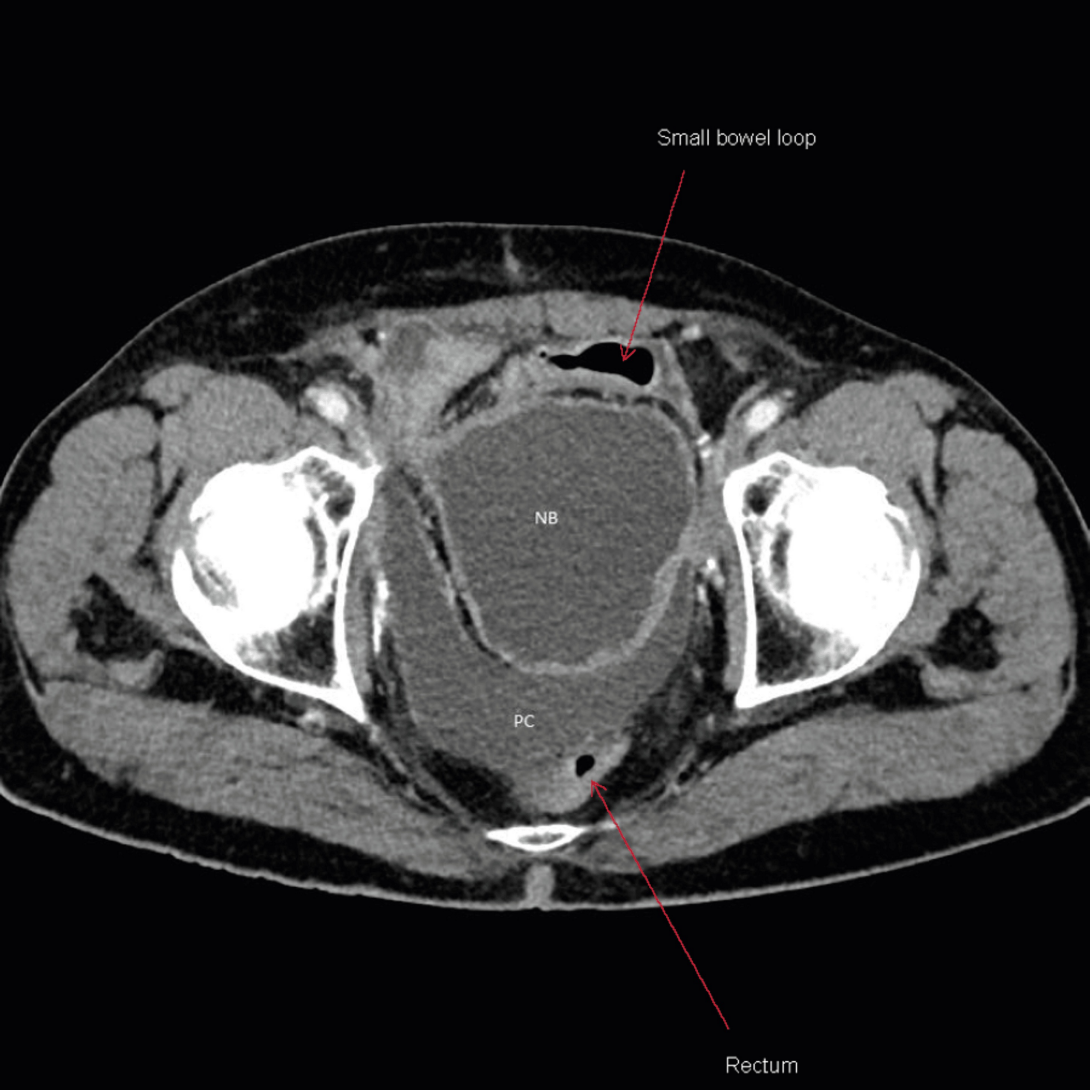
Axial view of contrast-enhanced computed tomography (CECT) performed on the day of admission showing pelvic fluid collection posterior to neobladder NB: Neobladder; PC: pelvic fluid collection

**Figure 2:**
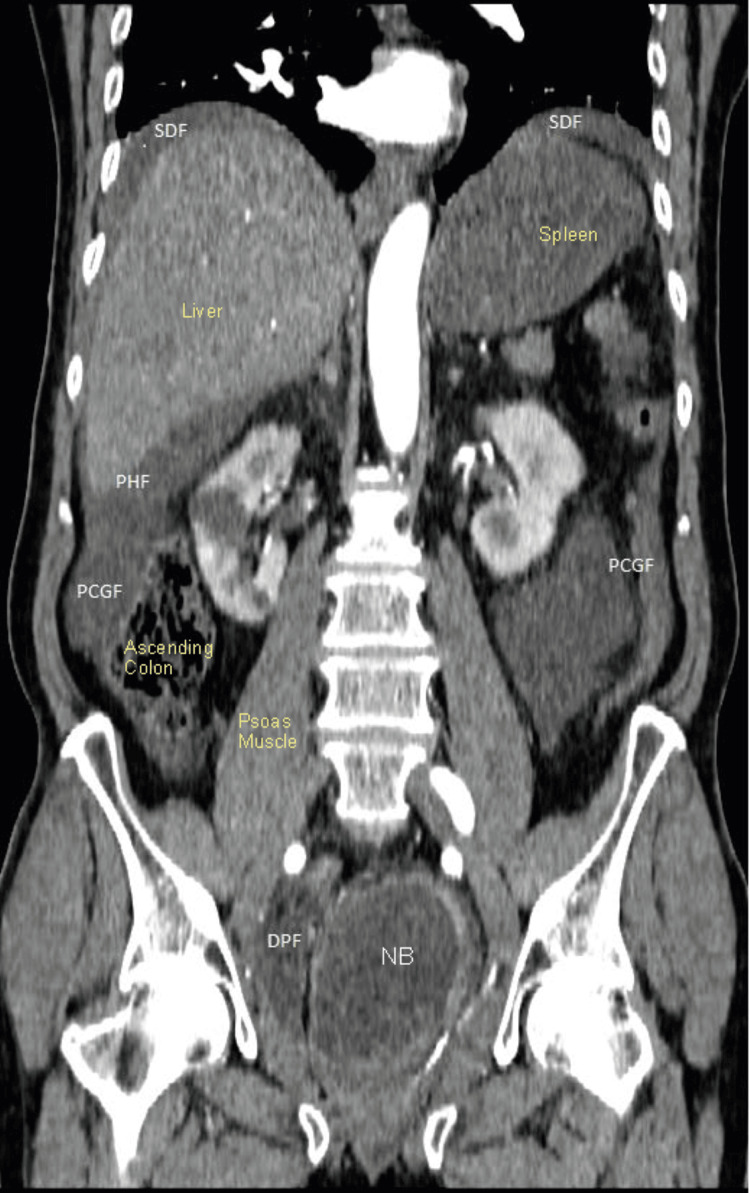
Coronal view of CECT of abdomen and pelvis performed on the day of admission showing moderate-volume free fluid in the deep pelvis, paracolic gutters, perihepatic, and subdiaphragmatic spaces. There was no pneumoperitoneum CECT: Contrast-enhanced computed tomography; SDF: subdiaphragmatic fluid collection; PHF: perihepatic fluid collection; PCGF: paracolic gutter fluid collection; DPF: deep pelvis fluid collection

Abdominal examination by the emergency physician revealed mild abdominal distension with some rebound tenderness on the lower abdomen but no guarding or rigidity. On auscultation, bowel sound was absent. Point-of-care ultrasound (PoCUS) at the emergency department revealed free fluid in the abdomen. After initial resuscitation and stabilization, the patient underwent contrast-enhanced computed tomography (CECT) abdomen and pelvis. CECT revealed moderate degree of free fluid in the whole abdomen without any free air (Figures 1-2).

Detailed history revealed that the patient is a musician by profession and lives alone. He underwent a robotic cysto-prostatectomy with bilateral pelvic lymph node dissection and orthotopic Ileal neobladder formation 12 months before this admission for muscle-invasive urothelial cancer of the bladder. He was fully continent and voiding spontaneously without elevated post-void residual (PVR) during follow-up. He had been under regular surveillance at the urology clinic, without evidence of disease recurrence. He was drinking alcohol at his home alone which resulted in a deterioration of his level of consciousness. Thus, he was unable to go to the toilet for voiding, and he was sleeping on the floor. He woke up early morning with abdominal pain and contacted an ambulance.

Following urology consultation, a diagnosis of spontaneous perforation of the neobladder was made. Expert advice was taken from a high-volume consultant urologist with extensive experience regarding the ongoing management of this patient.

After resuscitation, he was transferred to the intensive care unit (ICU) for close monitoring with a diagnosis of spontaneous perforation of the neobladder and sepsis. Conservative management continued with intravenous normal saline, tazobactam/piperacillin, and opiate analgesics with input from a multidisciplinary team (MDT). He responded slowly but steadily to resuscitation measures, and his urine output improved and vital signs stabilized.

**Figure 3:**
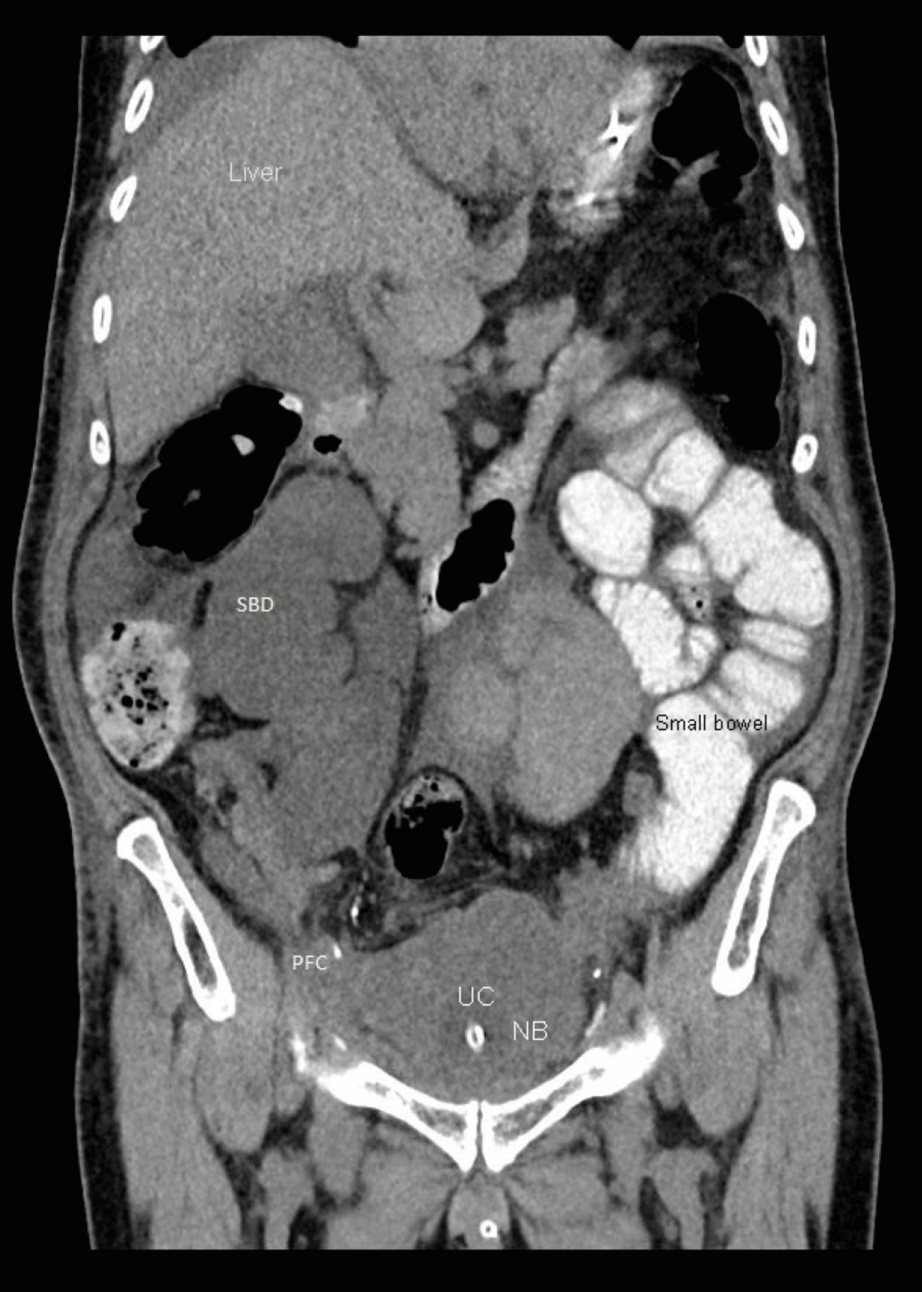
CT Abdomen and pelvis (coronal view) with intravenous and oral contrast and cystography phase three days after admission showing mild small bowel dilatation/distension and pelvic fluid collection SBD: Small bowel dilatation; PFC: pelvic fluid collection

**Figure 4:**
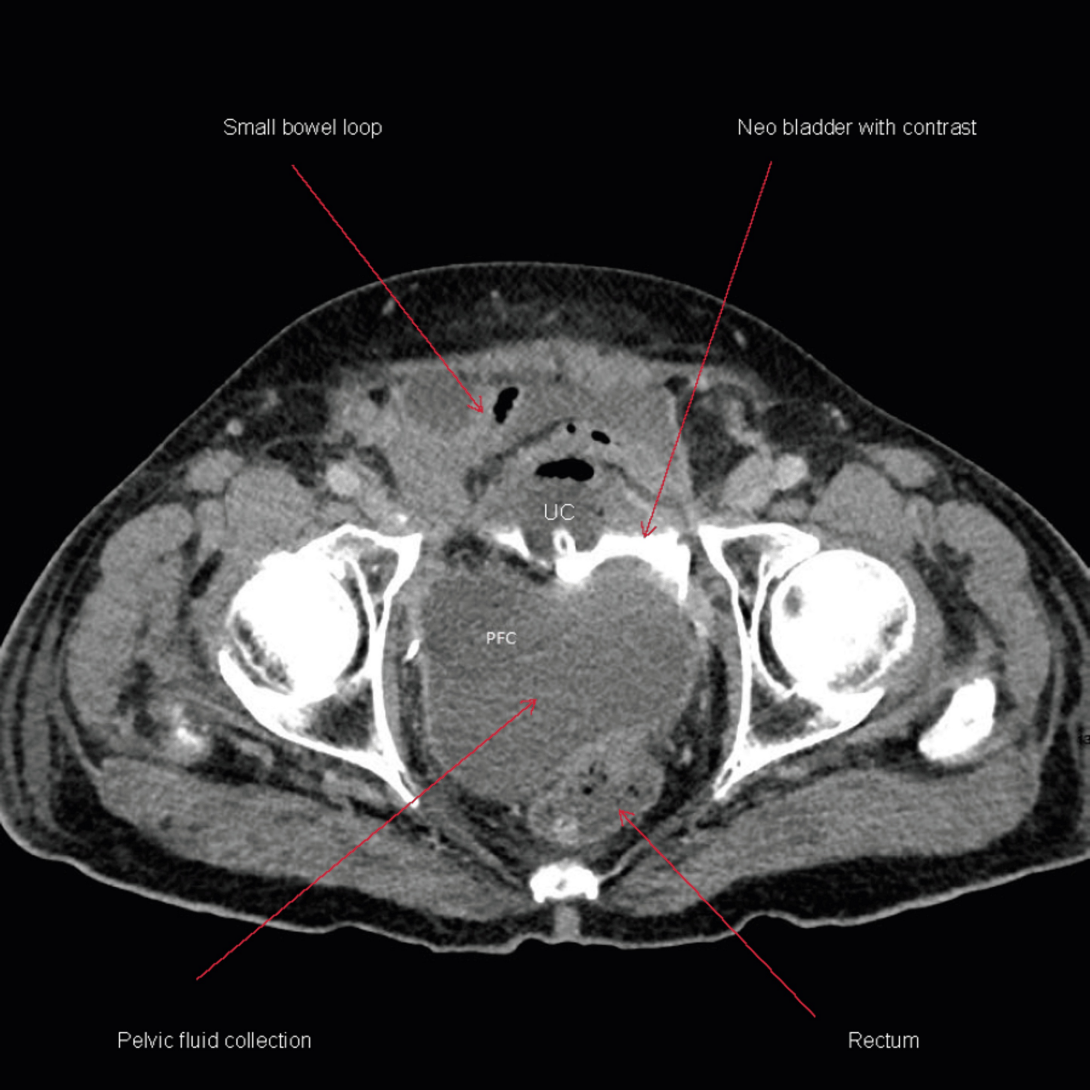
CT Abdomen and pelvis (axial view) with intravenous and oral contrast and cystographic phase three days after admission showing mild small bowel dilatation/distension and pelvic fluid collection PFC: Pelvic fluid collection

However, three days after admission, he developed moderate abdominal distension with mild deep tenderness but without any rebound tenderness or muscle guarding/rigidity. The peristaltic sound was not audible on auscultation. A repeat CT scan revealed mild small bowel distension likely from paralytic ileus as oral contrast was visible in the large bowel without any mechanical obstruction. However, a moderate-size fluid collection was observed in the pelvis posterior to the neobladder and anterior to the rectum (Figures 3-4). A likely explanation for this is that the perforation was in the posterior wall of the neobladder close to the urethra-neobladder anastomosis. A smaller amount of free fluid was also noted anterior to the neobladder, in the paracolic gutter, subdiaphragmatic space, and perihepatic space. A peripherally inserted central catheter (PICC) line was inserted for per-enteral feeding. Further advice was obtained from a high-volume urologist who advised to continue conservative measures.

**Figure 5:**
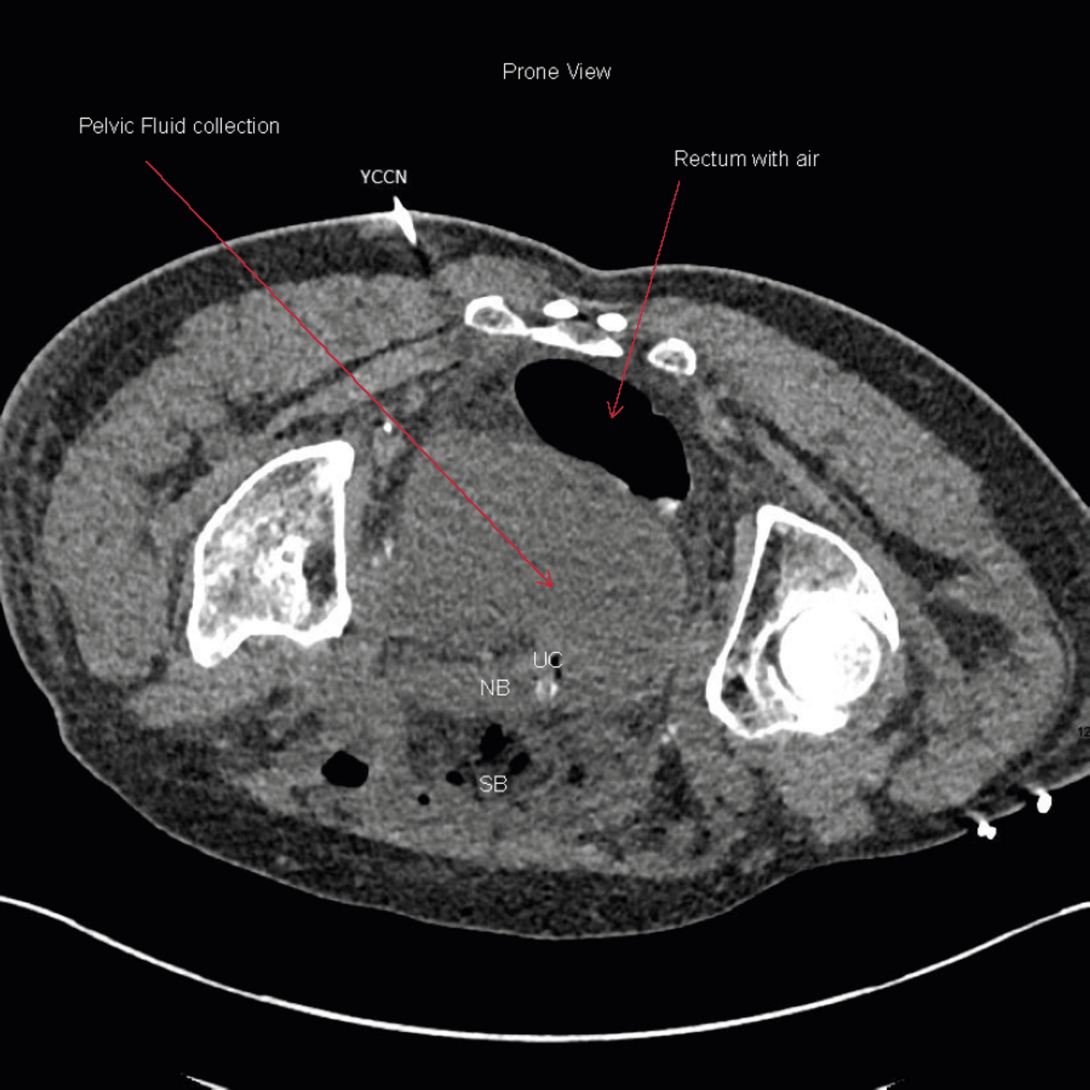
Axial prone CT view showing cutaneous entry path of CT-guided insertion of Yueh Centesis Catheter Needle (YCCN) (Cook Medical, Limerick, Ireland) to insert the pigtail drain into the pelvis

**Figure 6:**
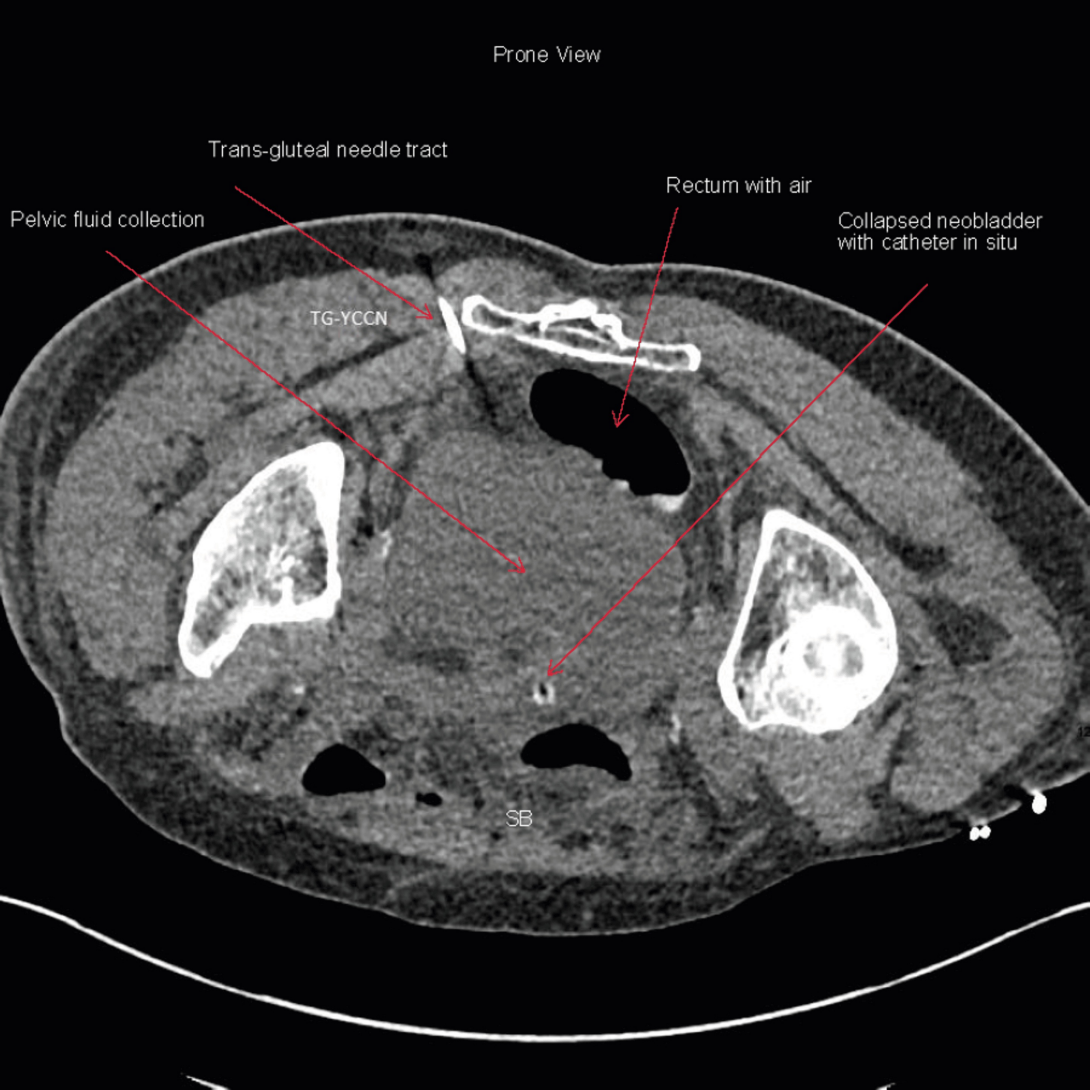
Axial prone CT showing trans-gluteal entry of CT-guided insertion of Yueh Centesis Catheter Needle (Cook Medical, Limerick, Ireland) to insert the pigtail drain into the pelvis TG-YCCN: Trans-gluteal Yueh Centesis Catheter Needle

A 15 cm, 5 Fr Yueh Centesis Catheter Needle (Cook Medical Europe, Limerick, Ireland) was inserted into the collection under a CT guide in a prone position through a trans-gluteal approach to avoid loops of bowel anteriorly (Figures 5-6). A pigtail drain is being inserted into the pelvic collection by an interventional radiologist. A 5 ml serosanguinous and 5 ml of serous fluid were aspirated and sent for culture and creatinine. Culture did not reveal any growth and elevated creatinine confirmed urine (Table 3).

**Table 3:** Pelvic fluid for creatinine 10 days post-admission

Pelvic fluid 10 days post-admission	Results
Drain fluid for creatinine	428 umol/L ( serum creatinine at the time 50 µmol/L)

**Figure 7:**
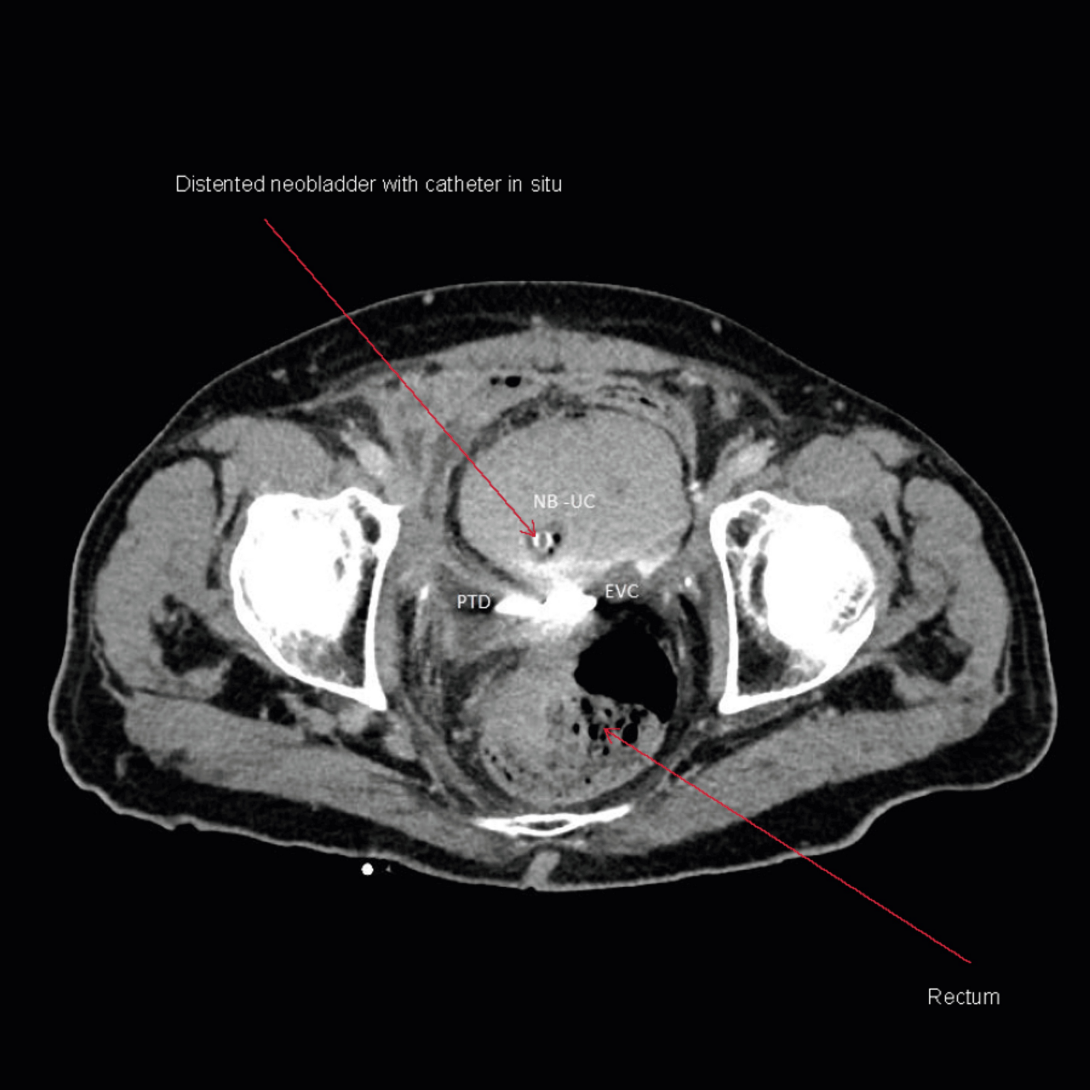
Axial view of CT cystogram two weeks post-admission and conservative management showing neobladder with urethral catheter in situ and minimal extravasation of contrast from the neobladder neck. Internal coiled end of the pigtail drain is visible as hyperdense shadow behind the neobladder. There is near complete resolution of pelvic collection NB-UC: Neobladder urethral catheter; PTD: internal end of pigtail drain; EVC: extravasation of contrast

Clinical and laboratory parameters continued to improve. Abdominal distension and tenderness improved gradually during the subsequent two weeks. Enteral feeding was commenced after two weeks. All blood parameters returned to normality. Repeat CT with intravenous contrast and cystographic phase 14 days after admission revealed a significant reduction in peritoneal free fluid and pelvic collection although a small leak was noted in the posterior wall of the neobladder near the anastomosis to the urethra with the internal end of pigtail drain was in place (Figure 7). CT cystogram was repeated after four weeks from the date of admission which revealed no further extravasation of contrast.

**Figure 8:**
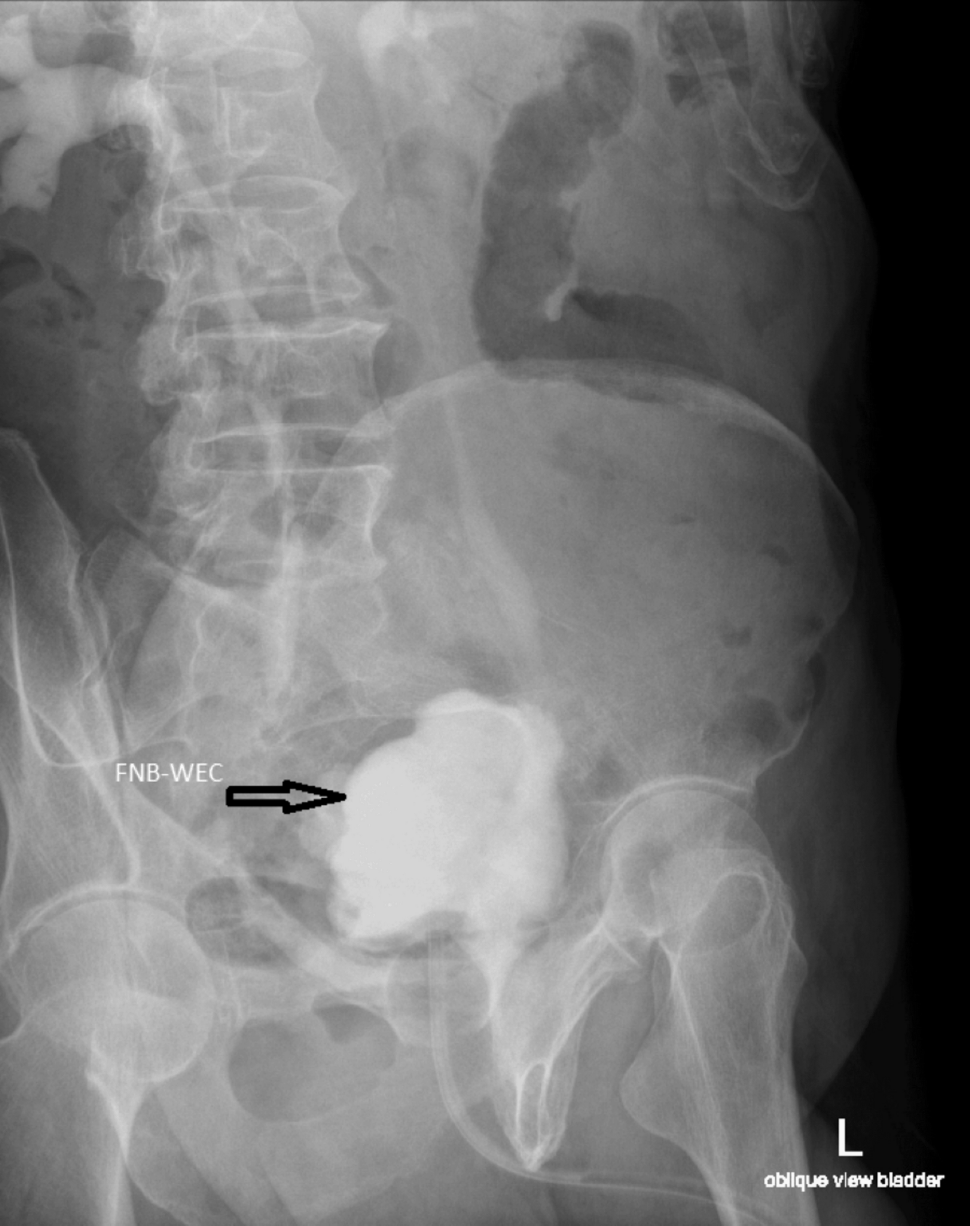
Cystogram prior to catheter removal showing filled neobladder without extravasation of contrast and free reflux of contrast into both ureters FNB-WEC: Filled neobladder without extravasation of contrast

**Figure 9:**
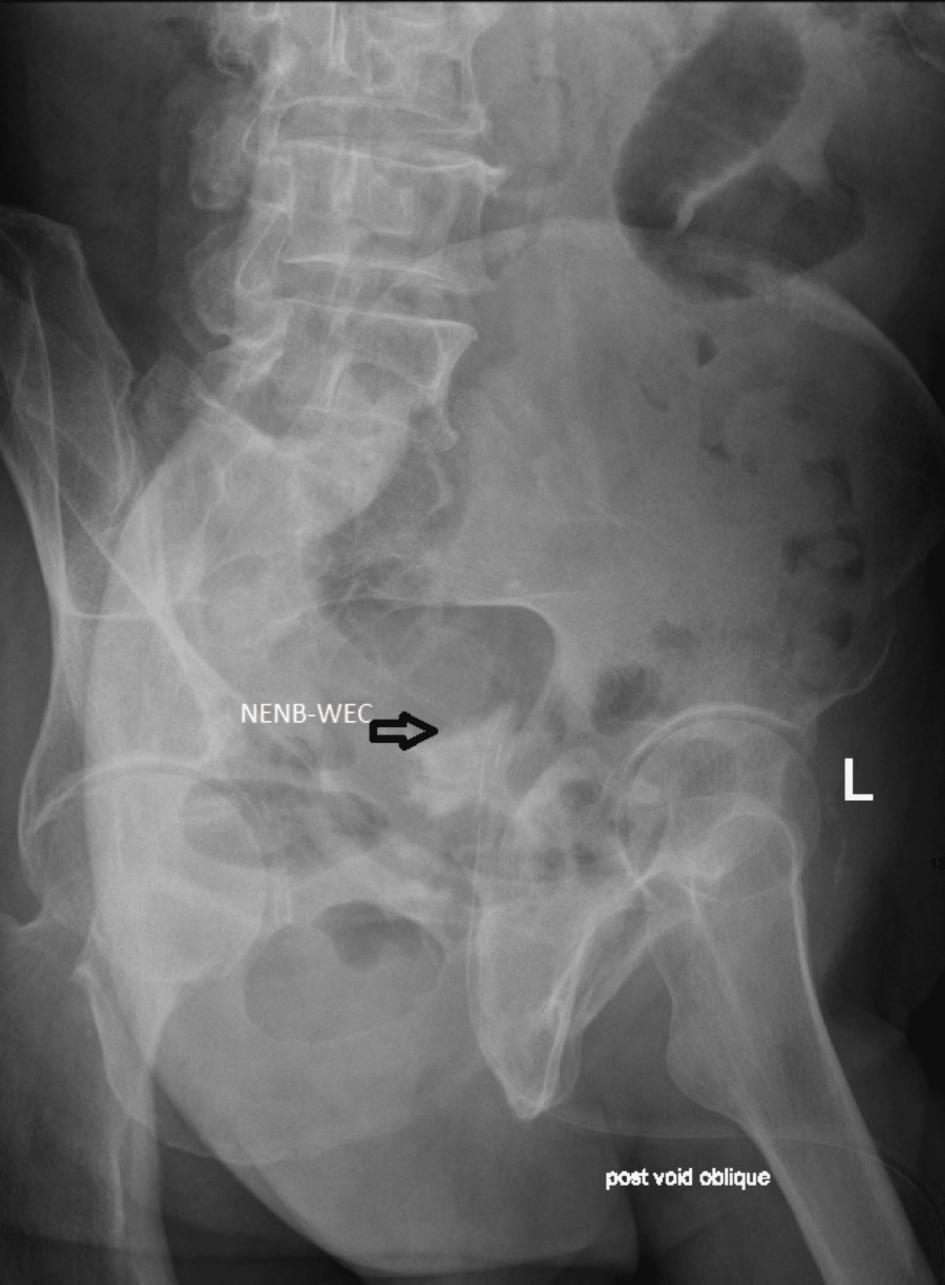
Cystogram prior to catheter removal showing near-empty neobladder without extravasation of contrast NENB-WEC: Near-empty neobladder without extravasation of contrast

The patient was managed successfully with conservative measures with input from an MDT including a microbiologist, radiologist, dietician, and ICU physician. As a precaution, the patient was discharged after five weeks with size 18 Fr two-way indwelling Foley-type all silicone catheter and leg bag with instruction on catheter care. At 10 weeks after admission, an outpatient cystogram was performed which did not demonstrate any leak (Figures 8-9). The catheter was then removed followed by Uroflow and PVR. His PVR was minimal and voiding spontaneously. His urine did not reveal any evidence of infection. His blood parameters were normal.

He was counseled extensively about the importance of self-care for neobladder including timed voiding, alcohol consumption, and intermittent self-catheter. He agreed to comply with our advice. The patient has preserved neobladder function and continence and is doing well until the last follow-up at six months post-discharge. He is scheduled for a yearly flexible cystoscopy for inspection of his urethra and neobladder and a CT scan as a part of ongoing surveillance for urothelial carcinoma of his bladder.

## Discussion

Our patient was managed conservatively (nonoperatively) with cautious active monitoring as the patient showed gradual and progressive improvement in the clinical condition after prompt catheter drainage and careful monitoring for urine output and other parameters. Repeat CT imaging after approximately 72 hours was performed to show radiological improvement. The patient was discharged once clinically well with an indwelling catheter, and the catheter was removed only after a cystogram had documented the reestablishment of urinary tract integrity with the absence of contrast leakage on an outpatient basis.

Diversion of urine with percutaneous nephrostomy may be indicated when there is hydronephrosis with elevated creatinine level with renal failure and sepsis which in turn may help in healing the rupture if the rent is small. For larger defects, it may not heal and may serve as a temporary diversion until the patient is clinically stable for a definitive repair. In case of associated intra-abdominal injury necessitating laparotomy, primary repair may be indicated. Our patient had elevated creatinine and some features of sepsis. Elevated creatinine was likely multifactorial including absorption of creatinine from the uroperitoneum (urinoma), urinary retention with backpressure effect, dehydration, and sepsis. Clinical and laboratory parameters improved steadily with conservative measures.

Spontaneous perforation of the neobladder is a rare but known serious complication. The etiology of perforation of the neobladder can be spontaneous, traumatic, infective, or iatrogenic. A review of current literature to date identified a total of 25 cases of spontaneous neobladder bladder perforation with six cases managed without operative intervention [8].

A total of 19 cases were reported between 1992 and 2013 and managed with surgical repair. Most reported individual cases with two authors describing a case series of five patients [9]. Nippgen et al. reported a series of five cases with spontaneous perforation of the neobladder [3]. 

The risk factors for spontaneous ileal neobladder perforation have been described in the literature. The two most common risk factors are overdistension of the neobladder and total continence [3]. Overexpansion of the neobladder due to urinary retention is considered a main factor in the pathogenesis. It is postulated that patients with total continence do not retain a 'pop-off' mechanism that causes leakage once neobladder pressure exceeds a certain threshold [3,8-10]. Our patient was totally continent and developed overdistension of the bladder from urinary retention.

The prognosis of these patients was highlighted as poor with a mortality rate of up to 80% in some series being reported [3]. Historically, in the case of neobladder perforation, immediate exploratory laparotomy with repair was often adapted in the past. Immediate exploratory laparotomy is not indicated for every case of spontaneous neobladder perforation. During the past four decades, there has been a shift from operative to nonoperative or conservative management of abdominal traumatic injuries especially for solid organs [5]. Increasingly intra-abdominal visceral solid organ injuries are being managed with a nonoperative or conservative approach with MDT involvement and cautious active monitoring. Immediate resuscitation using airway, breathing, and circulation (ABC) protocol, PoCUS, and CT scan once stable with MDT involvement and cautious active monitoring in the ICU environment can avoid emergency laparotomy. Furthermore, collaborative expert advice from high-volume specialist center can also help to guide management. Any deterioration in clinical condition should otherwise warrant exploratory laparotomy.

Traditionally, extraperitoneal bladder perforations are managed with a nonoperative approach, and intra-peritoneal bladder perforations are managed with surgical repair. It is possible that in our case, the size of the perforation was small, and thus, nonoperative management was successful. Nonoperative management of intra-peritoneal spontaneous neobladder perforation has been reported in the literature [8].

Delay in resuscitation and diagnosis is associated with considerable morbidity and mortality, emphasizing the importance of early diagnosis and prompt management of a neobladder perforation. Diagnosing a perforation of a neobladder requires a high index of clinical suspicion. A detailed history and an abdominal examination along CT scan usually unfold the diagnosis. If clinically indicated, a CT cystogram can provide an accurate diagnosis provided the patient is stable and there is no contraindication. CT cystogram is not essential to be performed immediately and can be delayed until the patient is stable. Emergency room physicians should be aware of such conditions and seek urgent urology consultation. Immediate catheter drainage of the neobladder is essential.

Being all alone while drinking a large amount of alcohol can be dangerous. Solitary binge drinking can result in fall or injury while intoxicated and would not have anyone present to help the person. In particular, for a person with a neobladder who requires timely voiding, this can have serious consequences. Binge drinking can potentially result in a reduced level of consciousness and urinary retention. A combination of the two is likely to result in spontaneous perforation of the neobladder. Our patient lives alone, and he was drinking alcohol at his home all alone which resulted in the deterioration of his level of consciousness. Thus, he was unable to go to the toilet for voiding, and he was sleeping on the floor. He woke up early hours of the morning with abdominal pain and then contacted an ambulance. At the emergency department, he received prompt resuscitation and imaging and then a referral to a urologist. Physicians in the emergency room should be aware of such an uncommon occurrence. He recovered successfully with a conservative nonoperative approach with the involvement of MDT and careful active monitoring. The patient was extensively counseled about preventative measures such as timed voiding, learning clean intermittent self-catheterization (CISC), and avoiding binge drinking.

## Conclusions

Spontaneous neobladder perforation can be successfully managed with a conservative nonoperative approach with cautious active monitoring involving an MDT and collaborative expert advice from a high-volume center. Conservative or nonoperative management has the potential to avoid an emergency exploratory laparotomy. However, if the patient’s symptoms and signs deteriorate clinically during conservative management, laparotomy and surgical repair of the defect are indicated. For neobladder perforation, the key to conservative management is prompt resuscitation, early cross-sectional imaging with CT, and a high index of suspicion. Immediate drainage of the neobladder with the correct placement of the urethral catheter is very important for the healing of the neobladder. Prompt referral to a urologist is also crucial. Patients with neobladder should be rigorously counseled about the importance of timed voiding, intermittent self-catheter, serious consequences of solitary binge drinking, and urinary retention.

## References

[1] Nam JK, Kim TN, Park SW, Lee SD, Chung MK (2013). The Studer orthotopic neobladder: long-term (more than 10 years) functional outcomes, urodynamic features, and complications. Yonsei Med J.

[2] Hautmann RE, de Petriconi RC, Volkmer BG (2011). 25 years of experience with 1,000 neobladders: long-term complications. J Urol.

[3] Nippgen JB, Hakenberg OW, Manseck A, Wirth MP (2001). Spontaneous late rupture of orthotopic detubularized ileal neobladders: report of five cases. Urology.

[4] Skrzynski CJ, Creswell KG (2021). A systematic review and meta-analysis on the association between solitary drinking and alcohol problems in adults. Addiction.

[5] Cioffi SP, Cimbanassi S, Chiara O (2023). Blunt abdominal trauma: watch and wait. Curr Opin Crit Care.

[6] Daniels R, Nutbeam T, McNamara G, Galvin C (2011). The sepsis six and the severe sepsis resuscitation bundle: a prospective observational cohort study. Emerg Med J.

[7] Dellinger RP, Levy MM, Carlet JM (2008). Surviving sepsis campaign: international guidelines for management of severe sepsis and septic shock: 2008. Crit Care Med.

[8] Nesbitt AL, Yuhico MP, Khan M (2022). Guideline proposal for the conservative management of a ruptured neobladder. J Clin Urol.

[9] Desgrandchamps F, Cariou G, Barthelemy Y, Boyer C, Teillac P, Le Duc A (1997). Spontaneous rupture of orthotopic detubularized ileal bladder replacement: report of 5 cases. J Urol.

[10] Månsson W, Bakke A, Bergman B (1997). Perforation of continent urinary reservoirs: Scandinavian experience. Scand J Urol Nephrol.

